# Complicated Post-Partum HELLP Syndrome Causing Acute Renal Failure and a Spontaneous Acute Subdural Hematoma

**DOI:** 10.7759/cureus.13233

**Published:** 2021-02-08

**Authors:** Farhan A Shah, Gilad Guez, Neil Patel, Brijesh B Patel

**Affiliations:** 1 Internal Medicine, Lewis Gale Medical Center, Salem, USA; 2 Internal Medicine, Edward Via College of Osteopathic Medicine, Blacksburg, USA; 3 Internal Medicine/Pulmonary and Critical Care, Lewis Gale Medical Center, Salem, USA

**Keywords:** hellp, acute subdural hematoma, acute renal failure and hemodialysis in icu

## Abstract

HELLP syndrome is characterized by hemolysis, elevated liver enzymes, and thrombocytopenia. It is a devastating illness that typically occurs in the third trimester of gestation. We present a unique case of complicated post-partum HELLP syndrome.

The patient was a 34-year-old Caucasian G1PO woman at 40 weeks’ gestational age who presented for induction of labor. She underwent successful vaginal delivery. However, postoperatively the patient developed HELLP syndrome complicated by acute renal failure. She was transferred to the intensive care unit, where her renal function continued to decline, ultimately necessitating hemodialysis. She subsequently spontaneously developed an acute subdural hematoma.

Most cases of HELLP syndrome occur in the third trimester, whereas fewer manifest post-partum. The pathophysiology of HELLP syndrome is poorly understood. While the defining organ of injury in HELLP syndrome is the liver, both kidney injury and spontaneous subdural hematomas can occur, as seen in this patient. The gold standard therapy for HELLP syndrome is prompt delivery of the fetus.

HELLP syndrome continues to be a serious constellation of symptoms that can affect women late in their gestational period. As illustrated in this case report, prompt diagnosis of HELLP syndrome and appropriate management is critical.

## Introduction

HELLP syndrome consists of hemolysis, elevated liver enzymes, and thrombocytopenia [1]. In its complete form, it is devastating to both the mother and fetus and is associated with complications including eclampsia, acute renal failure, cerebral hemorrhage, neonatal thrombocytopenia, and mortality of both the mother and fetus [1]. HELLP syndrome occurs in approximately 0.5% to 0.9% of all pregnancies, but primarily arises in the third trimester of gestation [1]. We present a unique case of a complicated post-partum HELLP syndrome in a previously healthy 34-year-old Caucasian G1PO woman, resulting in acute renal failure necessitating hemodialysis and a spontaneous acute subdural hematoma.

## Case presentation

The patient was a 34-year-old Caucasian G1PO woman at 40 weeks’ gestational age who presented for induction of labor. Initial vital signs and laboratory results were within normal limits. Labor was induced with oxytocin, and the patient had an epidural placed for anesthesia. She underwent successful vaginal delivery complicated by retained placenta. The patient developed persistent post-partum hemorrhage and the next day underwent uterine evacuation in the operating room, without any residual placental or uterine contents left. Postoperatively, the patient complained of a pressure-like pain in her lower back. She developed decreasing urine output despite adequate intravenous fluid administration, producing less than 50 milliliters of urine over the past 24 hours, as measured through her indwelling Foley urinary catheter. She was found to have several new abnormal laboratory test findings (Table 1), including leukocytosis of 21.57 k/uL, anemia with a hemoglobin of 8.1 g/dL, thrombocytopenia with platelets of 26 k/uL, a new acute kidney injury with BUN/Cr (blood urea nitrogen/creatinine) of 25/3.10 mg/dL, and significantly elevated liver function tests of AST/ALT (aspartate transaminase/ alanine transaminase) equal to 1,275/259 U/L. Lactate dehydrogenase was found to be severely elevated with a level of 3,916 U/L, and hemolysis was noted on peripheral blood smear.

**Table 1:** Laboratory tests performed during hospitalization --- Test not performed on that day of hospitalization

Test (reference value)	Day 1	Day 2	Day 3	Day 4	Day 5	Day 6	Day 7
White blood cells (4.50-10.50 k/uL)	8.7	21.57	24.41	26.04	23.69	20.79	19.82
Hemoglobin (11.4-15.5 g/dL)	13.8	8.1	7.1	7.0	6.5	7.1	8.6
Hematocrit (37.0-47.0%)	40.5	30.5	20.7	21.3	19.5	21.8	24.6
Platelets (130-385 k/uL)	143	26	76	80	99	102	115
Aspartate transaminase (15-37 U/L)	---	1275	1130	366	---	120	85
Alanine transaminase (13-61 U/L)	---	259	215	132	---	35	29
Total/direct bilirubin (0.20-1.00/0.00-0.20 mg/dL)	---/---	0.70/0.25	1.10/0.28	0.50/0.20	---/---	0.40/---	0.50/---
Creatinine (0.60-1.30 mg/dL)	---	3.10	3.50	5.50	5.20	4.40	6.60
Urea (7-18 mg/dL)	---	25	30	45	39	27	44
Lactate dehydrogenase	---	---	3916	---	2325	---	2120

**Figure 1:**
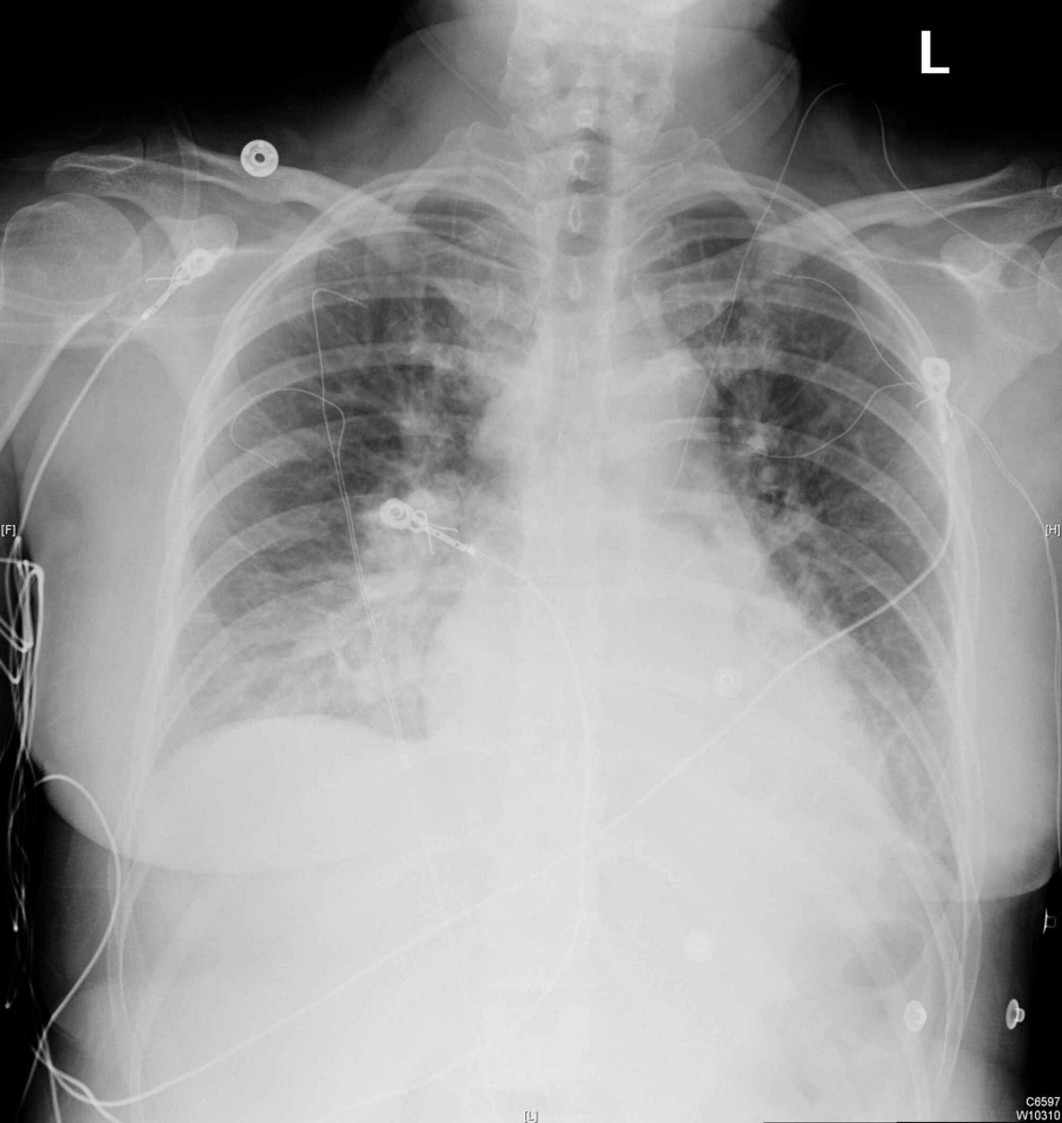
Chest radiograph demonstrating thickened interstitial markings, pulmonary edema, and bilateral pleural effusions

The patient was diagnosed with post-partum HELLP syndrome and transferred to the intensive care unit. She was transfused with packed RBCs and platelets accordingly. The patient then complained of a headache and had developed severe hypertension, necessitating intravenous antihypertensive therapy. Renal ultrasound revealed isoechoic kidneys compatible with medical renal disease and mild bilateral pelvocaliectasis. CT of the abdomen/pelvis revealed trace amounts of free abdominal/pelvic fluid. Chest radiograph (Figure 1) revealed vascular congestion and bilateral pleural effusions.

**Figure 2:**
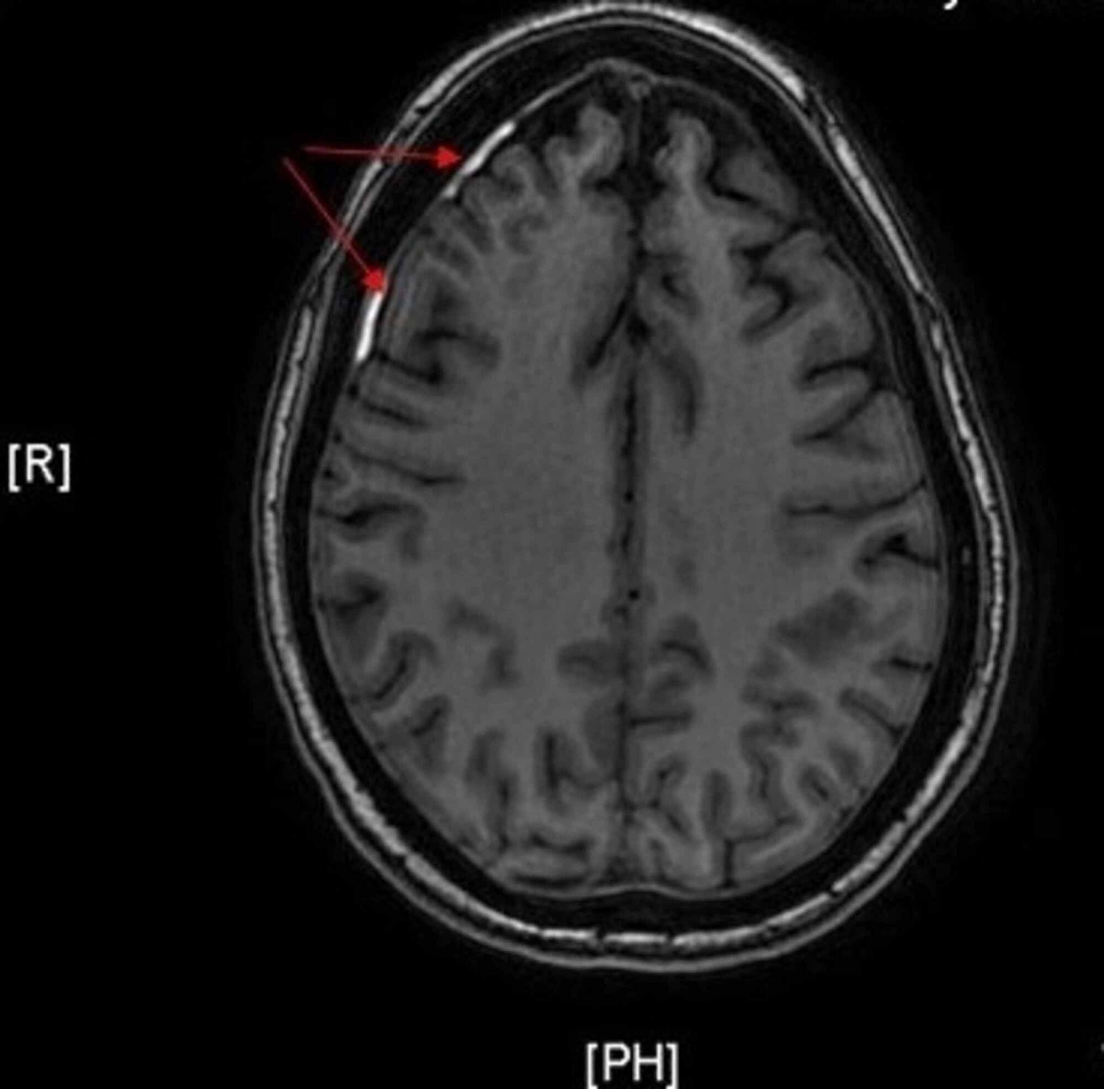
MRI of the brain with T1 hyperintensity (red arrows) consistent with a thin subdural hematoma, 3 mm in maximal thickness

Her renal function continued to decline. On the third postoperative day, the patient started her first session of hemodialysis, receiving a total of three sessions while hospitalized. During her last hemodialysis session, the patient complained of new-onset visual changes in her right eye and described seeing floaters. Subsequent MRI of the brain (Figure 2) revealed a subtle T1 hyperintensity consistent with an acute thin subdural hematoma. The patient was thereafter transferred to a different facility to undergo plasmapheresis.

## Discussion

HELLP syndrome was first identified as the constellation of clinical features consisting of hemolysis, elevated liver enzymes, and a low platelet count by Dr. Weinstein in 1982 [2]. It has been documented that HELLP syndrome occurs in approximately 0.5% to 0.9% of all pregnancies and can occur in a range of 10% to 20% of severe preeclampsia cases [1]. Most cases of HELLP syndrome occur in the third trimester. However, in approximately 11% of cases, the disease occurs at less than 27 weeks’ gestational age [3]. While around 69% of cases are antepartum, 31% of HELLP syndrome manifest post-partum, with most clinical signs and symptoms developing within the first two days but can manifesting up to seven days post-partum, as seen in this case report [3]. Risk factors for developing HELLP syndrome include multiparity, maternal age greater than 25 years old, white race, and history of poor pregnancy outcome [3]. The mortality rate for women diagnosed with HELLP syndrome has been documented to be around 1.1% [4].

The pathophysiology of HELLP syndrome is poorly understood but is thought to include several mechanisms of thrombotic microangiopathy, such as microvascular endothelial activation, cell injury, and thrombosis [5]. Another possible pathway is maternal rejection of the fetus as a product of physiological trophoblastic invasion coming into contact with immunocompetent maternal cells [6]. Other theories explore the roles of platelet plasminogen, inborn errors of fatty acid metabolism, and placenta-driven apoptosis of hepatocytes mediated by CD95/CD95-L [7-9]. Others interpret HELLP syndrome as simply a more severe and complicated form of preeclampsia; however, 20% of HELLP syndrome patients do not have signs of preeclampsia, such as hypertension or proteinuria, and tend to have higher levels of HLA-DR (human leukocyte antigen - DR isotype) and some inflammatory markers, such as C-reactive protein, when compared to typical preeclampsia [10].

While the defining organ of injury in HELLP syndrome is the liver, kidney injury occurs in roughly 50% of patients, but the etiology and mechanism of injury is poorly understood as well. Some research suggests kidney injury is secondary to both preeclampsia and acute tubular necrosis [11]. Histological findings tend to be similar to thrombotic microangiopathy such as endothelial swelling and occlusion of capillary lumens, with the notable lack of luminal thrombi [11]. The presence of thrombi does increase the incidence of severe HELLP syndrome [11].

In addition to kidney injury, complications of HELLP syndrome can involve many other organ systems. Subcapsular hematomas are a known complication and may result in the life-threatening complication of spontaneous hepatic rupture, which has a mortality rate of up to 50% [12]. Other maternal complications include eclampsia, disseminated intravascular coagulation, placental abruption, severe ascites, cerebral infarction and hemorrhage, brainstem hemorrhage, and cerebral edema, and maternal death [1]. Complications affecting the fetus include neonatal thrombocytopenia, respiratory distress syndrome, intrauterine growth restriction, and perinatal death [1]. Also, potentially permanent vision loss can occur due to hemorrhagic and vaso-occlusive retinopathy similar to Purtscher retinopathy [11].

Initial clinical presentation of patients with HELLP syndrome often includes abdominal and /or epigastric pain, nausea, vomiting, and malaise [12]. Occasionally, patients may also present with ascites, jaundice, and oliguria, but these findings are typically less common. Initial assessment may also yield hypertension along with proteinuria, as seen in up to 80% of patients [12].

Definitive diagnosis is dependent on the presence of the following laboratory findings: demonstrable evidence of hemolysis (with either the presence of schistocytes on blood smear, elevated bilirubin greater than or equal to 1.2 mg/dL, elevated serum lactate dehydrogenase greater than or equal to 600 IU/L), thrombocytopenia of less than or equal to 100 k/uL, and AST greater than or equal to 70 IU/L [12]. Patients with some but not all of these findings are given the diagnosis of partial HELLP syndrome. Coagulation studies may be abnormal as well, particularly elevations in prothrombin time and partial thromboplastin time, but these derangements are usually mild if seen [12].

The main differential diagnosis of HELLP syndrome is severe preeclampsia, as both conditions have a large overlap of presentation and symptomatology, but severe preeclampsia will not have the aforementioned laboratory findings. Other differentials include acute fatty liver of pregnancy (more commonly associated with PT/PTT (prothrombin time/partial thromboplastin time) derangements and liver failure), thrombotic thrombocytopenic purpura, and hemolytic uremic syndrome (both of which typically do not include derangements in hepatic function) [12].

Initial management of HELLP is to treat mothers who are unstable and assess fetal viability. Mothers with severe hypertension, an unstable fetus, or epigastric pain should be assessed and tended to urgently. Those with severe hypertension can require pharmacological antihypertensive treatment. Women who present with epigastric or right upper quadrant pain need to be carefully examined for a hepatic bleed or hemorrhage. Patients in this setting may also experience shoulder or back pain, dyspnea, or nausea and vomiting [13,14]. HELLP patients experiencing similar symptoms revealed abnormal findings on liver imaging, with the most common being a hematoma or hemorrhage [13]. In those with hepatic bleeding, the correlation between aminotransferase levels and liver histology is weak [15]. Therefore, those with symptoms should undergo hepatic imaging regardless of lab values [16]. Bedside ultrasonography is preferred for initial imaging, which can then be followed up with CT or MRI imaging especially when management decisions are needed.

Imaging that reveals a subcapsular hematoma of the liver is especially concerning, as rupture can be fatal for both the mother and fetus. If a hepatic subcapsular hematoma is evident, volume replacement and transfusion is necessary [17]. Prompt delivery of the fetus should also be prioritized once the mother is stable. For a ruptured hepatic subcapsular hematoma, operative management will be required by trauma surgeons [17].

The gold standard therapy for HELLP, regardless of the conditions during pregnancy, is prompt delivery after the mother has been stabilized. This approach has been proven to be the most effective treatment, especially in those presenting after 34 weeks’ gestation, fetal demise, or abruptio placentae [18,19]. If a mother is not presenting in these conditions, a course of corticosteroids is recommended for 48 hours before delivery is indicated [18]. Magnesium sulfate is indicated for those presenting between 24 and 32 weeks of pregnancy for nervous system protection [18]. Antihypertensive medication should be given for severe hypertension, and RBCs can be transfused if hemoglobin levels fall below 7 g/dL. Platelet transfusions should be pursued in any patient that is having an acute and active bleed and has thrombocytopenia [18]. During deliveries, the platelet count should be maintained above 20,000 cells/microL to avoid bleeding [18]. This threshold varies, though, according to experts and can depend on multiple factors. For the route of delivery, vaginal delivery is preferred if the fetus is stable. In a prospective, double-blind clinical trial that included 132 women with HELLP syndrome, dexamethasone treatment was not found to improve the outcome of women with HELLP syndrome, with no significant differences found in the time to recovery of platelet levels, lactate dehydrogenase, aspartate aminotransferase, and the development of complications [20].

## Conclusions

HELLP syndrome continues to be a devastating constellation of symptoms that can affect women late in their gestational period. However, given the chance of post-partum HELLP syndrome in otherwise healthy women with normal gestation, clinicians should remain cognizant of this contingency, continue to scrutinize and monitor the mother's vitals throughout the peripartum period, and diagnose and treat HELLP syndrome early, while educating their patients and themselves accordingly.
